# Overexpression of *PavHIPP16* from *Prunus avium* enhances cold stress tolerance in transgenic tobacco

**DOI:** 10.1186/s12870-024-05267-2

**Published:** 2024-06-12

**Authors:** Runrun Yu, Qiandong Hou, Hong Deng, Ling Xiao, Xiaowei Cai, Chunqiong Shang, Guang Qiao

**Affiliations:** Key Laboratory of Plant Resource Conservation and Germplasm Innovation in Mountainous Region (Ministry of Education), College of Life Sciences, Institute of Agro-bioengineering, Guizhou University, Guiyang, 550025 Guizhou Province China

**Keywords:** Sweet cherry, HIPP, Genetic transformation, Low-temperature stress, Protein interaction

## Abstract

**Background:**

The heavy metal-associated isoprenylated plant protein (HIPP) is an important regulatory element in response to abiotic stresses, especially playing a key role in low-temperature response.

**Results:**

This study investigated the potential function of PavHIPP16 up-regulated in sweet cherry under cold stress by heterologous overexpression in tobacco. The results showed that the overexpression (OE) lines’ growth state was better than wild type (WT), and the germination rate, root length, and fresh weight of OE lines were significantly higher than those of WT. In addition, the relative conductivity and malondialdehyde (MDA) content of the OE of tobacco under low-temperature treatment were substantially lower than those of WT. In contrast, peroxidase (POD), superoxide dismutase (SOD), catalase (CAT) activities, hydrogen peroxide (H_2_O_2_), proline, soluble protein, and soluble sugar contents were significantly higher than those of WT. Yeast two-hybrid assay (Y2H) and luciferase complementation assay verified the interactions between PavbHLH106 and PavHIPP16, suggesting that these two proteins co-regulated the cold tolerance mechanism in plants. The research results indicated that the transgenic lines could perform better under low-temperature stress by increasing the antioxidant enzyme activity and osmoregulatory substance content of the transgenic plants.

**Conclusions:**

This study provides genetic resources for analyzing the biological functions of *PavHIPP*s, which is important for elucidating the mechanisms of cold resistance in sweet cherry.

**Supplementary Information:**

The online version contains supplementary material available at 10.1186/s12870-024-05267-2.

## Background

The heavy metal-associated (HMA) genes are an important family of proteins for maintaining heavy metal homeostasis and regulating low-temperature response in plants. These proteins often comprise one or more HMA structural domains, approximately 70 amino acids in length [1, 2]. HMA proteins in plants constitute a large family that includes HIPPs [3, 4]. HIPPs usually consist of one or two conserved metal-binding HMA structural domains and isoprenylated motifs [5]. The isoprenylated motifs encompass the conserved Cys-x-x-Cys (CxxC, x being any amino acid) motif, in which the sulfate group of the Cys residue acts as a ligand for binding metal ions, possibly by participating in the binding to other proteins [6]. Although the HMA domains and the isoprenylated motifs are prevalent in a wide variety of organisms, their functional interactions in the same protein are predominantly observed in vascular plants [4]. For example, in *Arabidopsis thaliana*, acyl-CoA-binding protein 2 (ACBP2) interacts with farnesylated protein 6 (AtFP6), which has a metal-binding motif (M/LXCXXC), and functions by binding Cd^2+^, Cu^2+^, and Pb^2+^ [7].

Current research on HIPPs has only concerned their involvement in regulating metal homeostasis in plants and the detoxification of heavy metal toxins accumulated in plants [8–10]. Heterologous expression of the *HIPP20*/*22*/*26*/*27* of *Arabidopsis thaliana* in yeast, enhances the Cd resistance of the Cd-sensitive yeast strain *ycf1*, suggesting a possible Cd detoxification effect role of HIPPs [4]. HIPPs have been reported to be up-regulated in response to abiotic stresses such as low temperature, drought, and salt stress, thus alleviating them [11]. HIPPs are also involved in plant seed development and flowering processes [12]. For example, *Arabidopsis AtHIPP26* is induced by low temperature and drought and is down-regulated during abscisic acid (ABA) treatment, leaf development, and senescence. It also interacts with the zinc-finger homology structural domain transcription factor *ATHB29*, which downstream regulates drought-responsive genes [3]. Additionally, the AtHIPP3 protein binds with the zinc finger domain to inhibit flowering and is also induced under biotic stresses in *Arabidopsis thaliana* [3, 12, 13], whereas *OsHIPP24* reduces rice plant height [14]. In addition, HIPPs also play a role in plant hormone signaling pathways, for example, *Arabidopsis* HIPP proteins regulate cytokinin responses and thus plant root development [15].

Sweet cherry (*Prunus avium* L.) belongs to the Rosaceae, *Prunus* fruit tree. Sweet cherry is a colorful fruit containing rich vitamins and other nutrients, and has high food and economic values [16]. Its flower and fruit development are very susceptible to chill, freezing weather, leading to significant yield and quality declines [17]. Therefore, studying the sweet cherry’s response to low-temperature stress and its regulatory pathway is very important for cultivating sweet cherry varieties with excellent cold tolerance, as the loss imposed by low temperatures can be effectively reduced.

The expression of C repeat binding factor *1* (*CBF1*), *CBF2*, and *CBF3* in *Arabidopsis* appeared to be altered in response to short periods of low-temperature induction [18], whereas sustained low temperatures indicated that CBFs regulated the up-regulation of various target genes [19]. Seed germination is the initial and critical biological stage of the life cycle of many flowering plants and is extremely important for plant growth and development [20]. Low-temperature stress also affects plant morphology. For example, plant leaf structure and stomatal size are affected by low-temperature stress, resulting in stomatal closure [21]. A suitable temperature is one of the most important environmental factors for plant growth and development, and plants suffering from low-temperature damage often experience growth slowdown or even death. Low-temperature stress also affects various plant physiological indexes to different degrees, where the soluble sugar content increases to protect the sensitive components and reduce injury. Increases in osmoregulatory substances, such as proline and soluble protein content, help maintain cold-induced enzyme conformation changes in plants [22]. Low temperatures cause reactive oxygen species (ROS) accumulation as well, such as H_2_O_2_ production, resulting in severe cellular damage, whereas plants stimulate the POD, SOD, and CAT activity to degrade ROS, thus protecting the cells from the attack while allowing quick plant recovery [23].

Previously, PavHIPP16 was screened by Y2H to interact with PavbHLH106, and it was found that *PavbHLH106* overexpression enhanced the tolerance of tobacco to cold stress [24]. Therefore, it is important to study *PavHIPP16* to enhance the low-temperature tolerance of plants, but the role of HIPPs in low-temperature stress and their regulatory mechanisms are still unknown. This study verified the function of the *PavHIPP16* gene by genetically transforming tobacco, and the results indicated its positive role in tobacco low-temperature response. The interaction of PavHIPP16 with PavbHLH106 was verified by Y2H and luciferase complementation assay. The findings elucidated the molecular mechanism underlying the cold resistance of tobacco mediated by the *PavHIPP16*. These insights could contribute to the genetic resources for anti-cold molecular breeding.

## Results

### Sequence analysis of ***PavHIPP16***

**Fig. 1:**
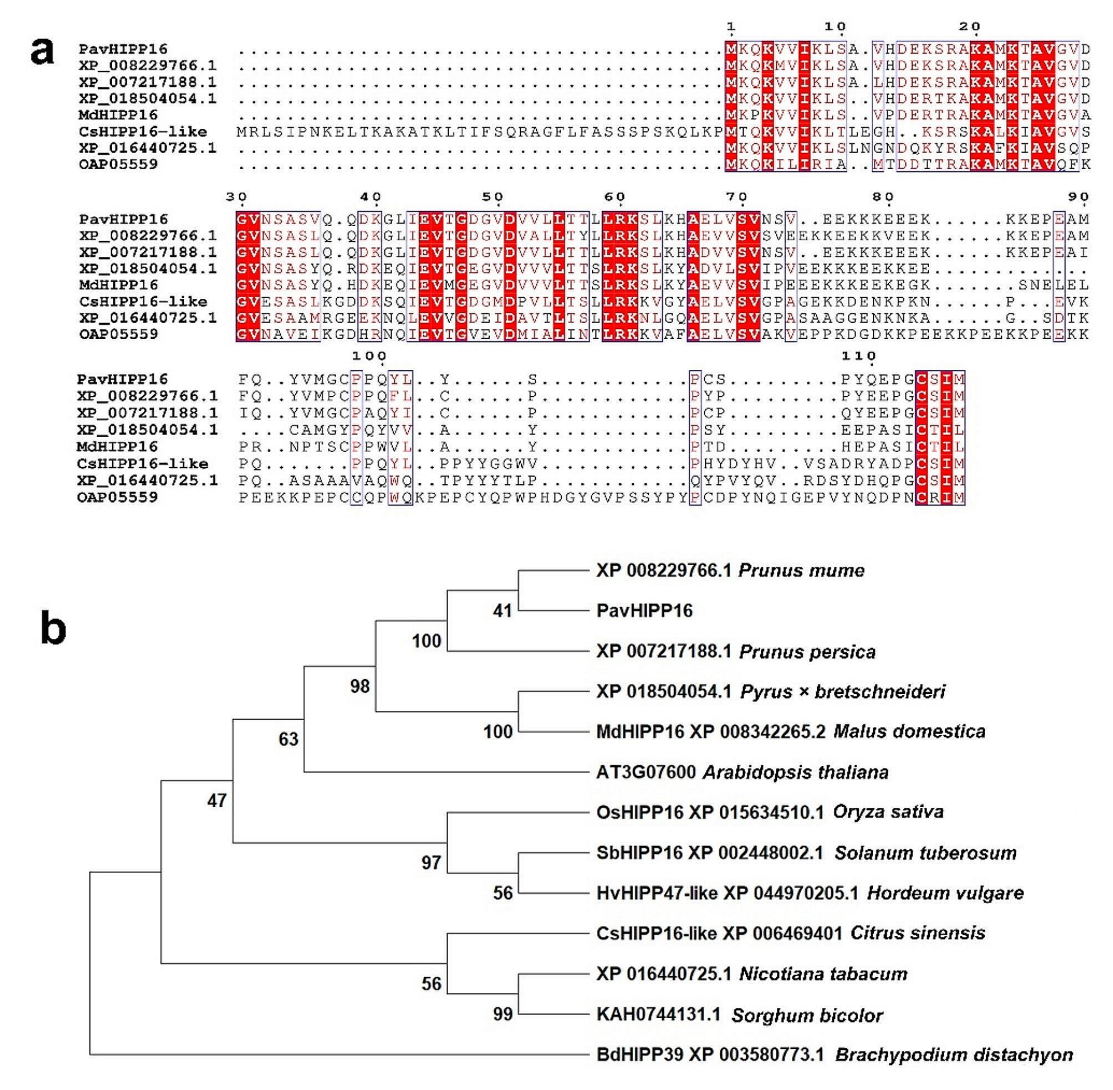
Sequence analysis and phylogenetic relationships of PavHIPP16. (**a**) Multiple sequence comparison of PavHIPP16 with homologous proteins from other plant species. The red background indicates that the amino acids at this position are fully conserved. (**b**) Phylogenetic tree constructed in MEGA11 using the NJ method and setting Bootstrap to 1000. Species include sweet cherry, Chinese plum, peach, white pear, apple, *Arabidopsis*, rice, potato, barley, sweet orange, tobacco, sorghum, and *Brachypodium distachyon*

HIPPs are ubiquitous and conserved in vascular plants. To understand the sequence characteristics, the protein sequence of PavHIPP16 was compared and analyzed for evolutionary relationships. Multiple sequence alignment showed (Fig. 1a), that PavHIPP16 was conserved with homologous amino acid sequences from other species, suggesting that the gene may have a similar function across different plants. Evolutionary analysis was performed to investigate the homology of PavHIPP16 with sequences from other species, including selected homologous proteins from dicotyledon and monocotyledon plants (Fig. 1b). The results indicated that PavHIPP16 clustered with homologous proteins from dicotyledonous plants within the Rosaceae family, consistent with the classification of Rosaceae.

### Acquisition and characterization of tobacco overexpressing ***PavHIPP16***

Based on the PCR amplification of the open reading frame (ORF) sequence of *PavHIPP16*, the 354 bp coding sequence (CDS) of *PavHIPP16* was cloned from sweet cherry leaves (Figure S1). Then, WT tobaccos were genetically transformed using the “leaf disk method” to obtain resistant seedlings (Figure S2).

**Fig. 2:**
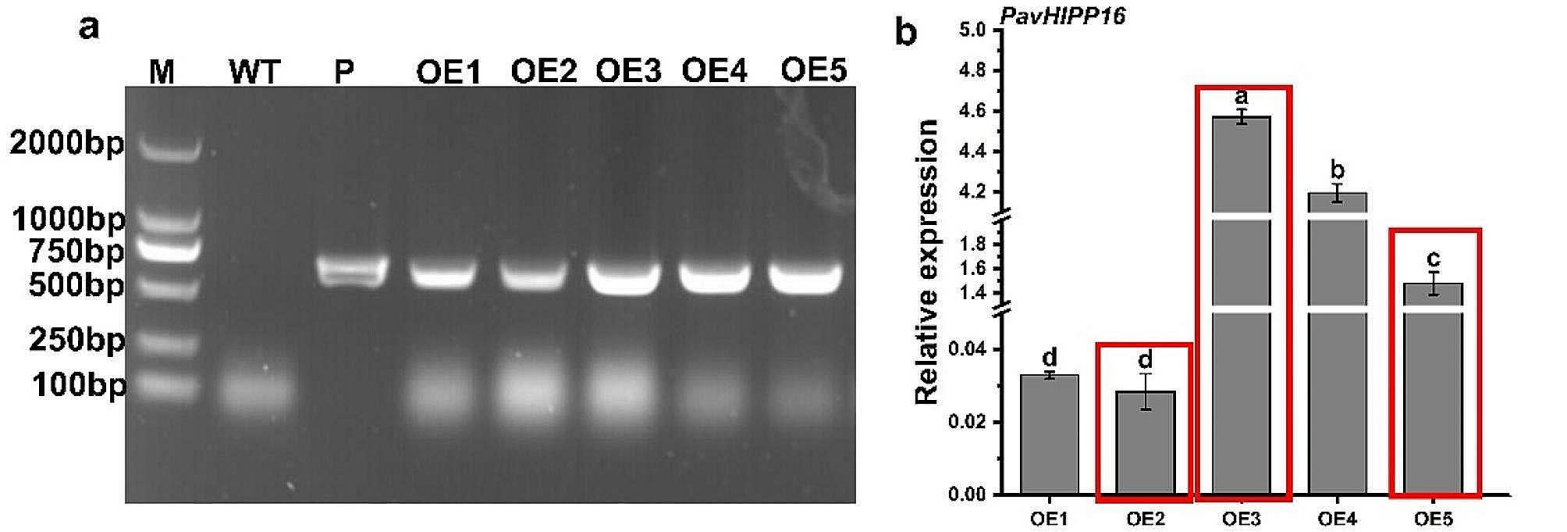
PCR validation and expression analysis of *PavHIPP16* genetically transformed tobacco. (**a**) Specific primers for PCR validation of the transgene and WT. M is the Marker for D2000 and P indicates recombinant plasmid. (**b**) Expression level of *PavHIPP16* in transgenic plants (Red boxes indicate overexpression plants for subsequent experiments). Data are shown as mean ± SE of three independent experiments (*n* = 3, biological replicates), with different letters representing significant differences at various levels (***P*** **< 0.05**)

The extracted tobacco gDNA was verified by PCR amplification, and five transgenic-positive resistant seedlings were obtained (Fig. 2a). *PavHIPP16* expression was determined by qRT-PCR, and *PavHIPP16* expression differences were found among the five lines (Fig. 2b), with OE3 exhibiting the highest expression level, and OE2 showing the lowest expression level. Three overexpressing plants were selected for further function investigation.

### Overexpression of ***PavHIPP16*** promoting germination and root elongation in tobacco

**Fig. 3:**
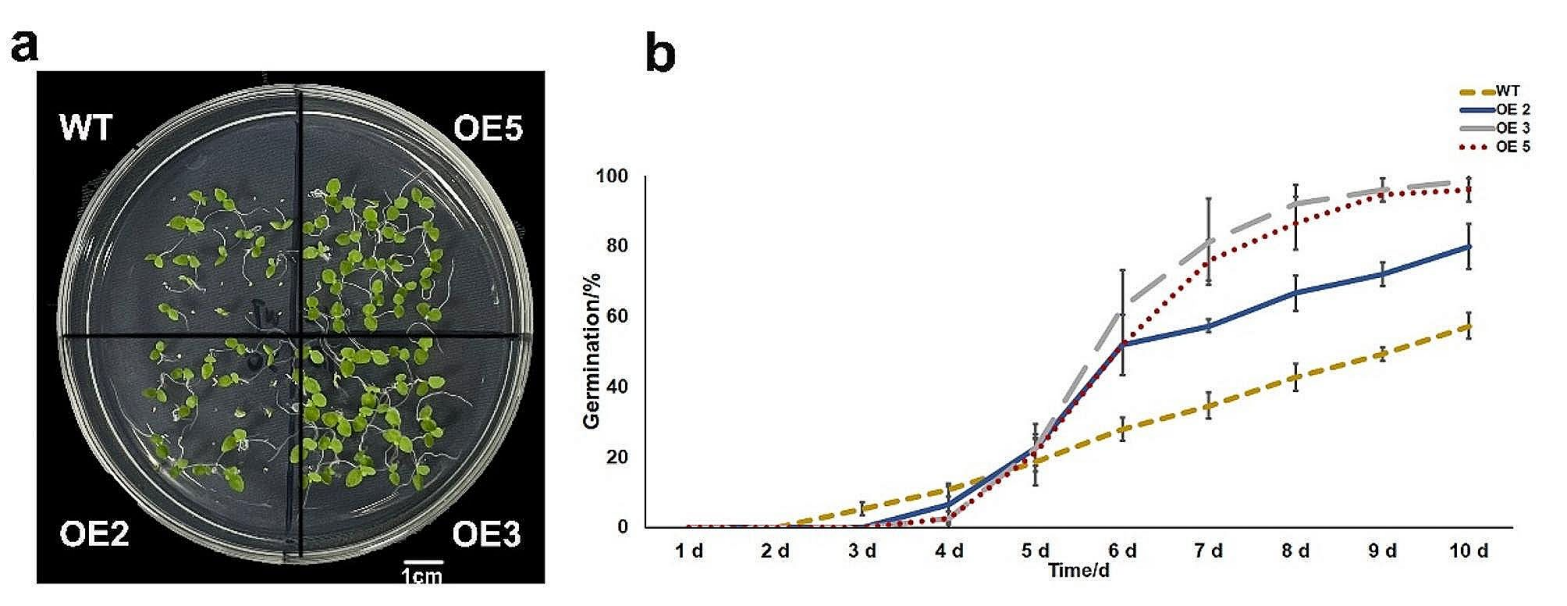
Analysis of germination rate of *PavHIPP16* transgenes tobacco. (**a**) Germination of tobacco OE and WT lines after 10 d of growth; (**b**) Germination statistics of tobacco seeds grown in MS medium for 10 d. Different colors are used to distinguish different tobacco lines. Data are shown as mean ± SE of three independent experiments (*n* = 3, biological replicates)

The OE lines and WT seeds were placed in the medium to observe their germination rates (Fig. 3). The results showed that the germination of WT tobacco seeds was earlier than that of OE, with WT seeds starting at 2 d and OE after 3 d. In terms of germination rate, that of WT tobacco at 10 d averaged 57.3%, whereas those of OE2, OE3, and OE5 averaged 80%, 98.7%, and 96%. The seed germination rates of the OE lines were significantly higher than that of WT.

**Fig. 4:**
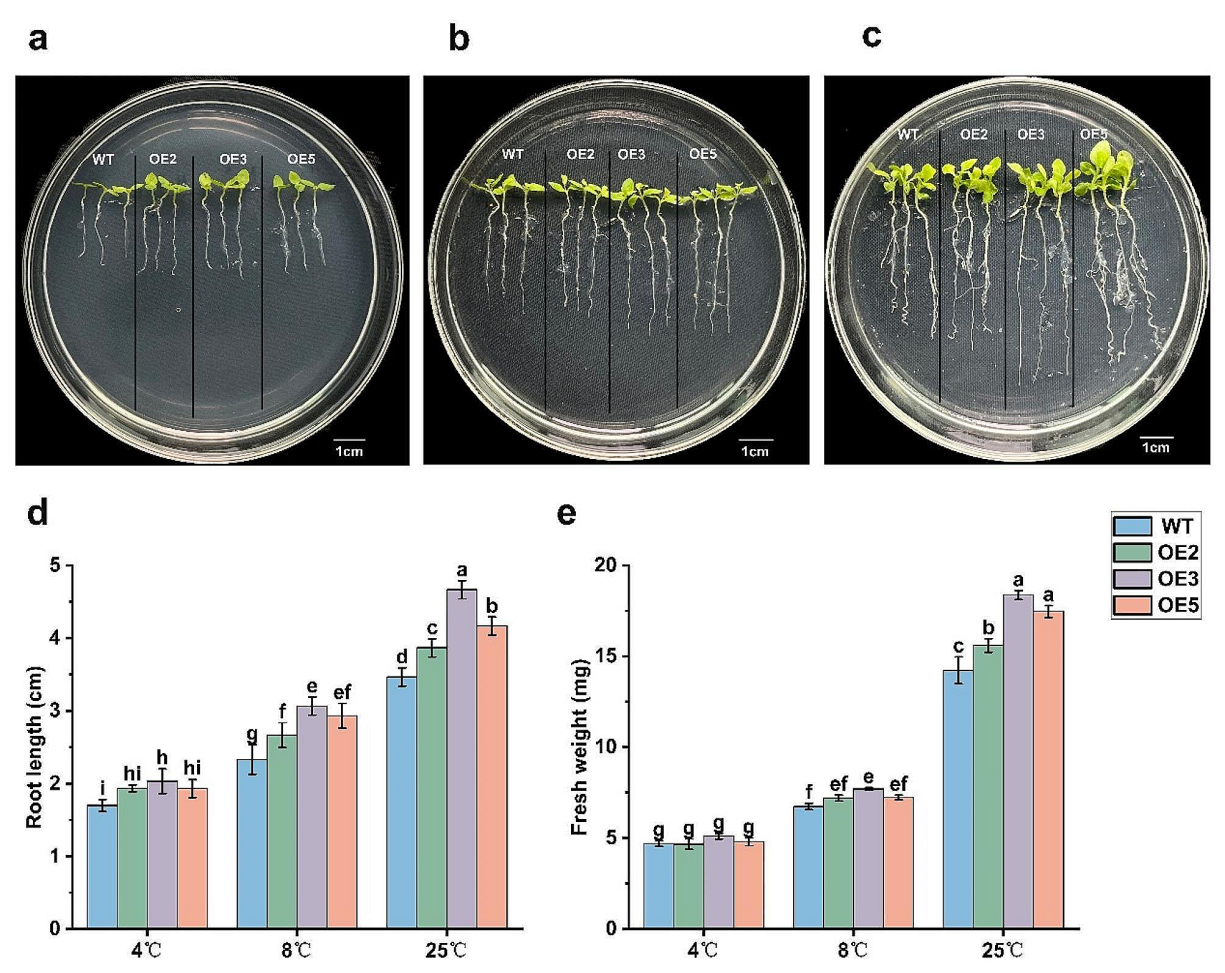
Root length and fresh weight of tobacco under low-temperature treatment. (**a-c**) Root length of OE2, OE3, OE5, and WT tobacco under low-temperature stress (a: 4 °C, b: 8 °C, and c: 25 °C); (**d**) Root length statistics after low-temperature treatment. (**e**) Comparison of fresh weight after low-temperature treatment. Data are shown as mean ± SE of three independent experiments (*n* = 3, biological replicates), with different letters representing significant differences at different levels (***P*** **< 0.05**)

After culturing for 10 d, The OE and WT tobacco seedlings were transferred to a new medium for low-temperature stress treatment (Fig. 4). According to the observation, the growth of OE seedlings was significantly better than WT in terms of root length and fresh weight, suggesting that the enhanced tolerance of tobacco to low temperatures was attributed to the well-developed root systems due to *PavHIPP16* overexpression, which efficiently absorbed nutrients and water.

### Effect of low-temperature stress on stomata of tobacco overexpressing the ***PavHIPP16***

**Fig. 5:**
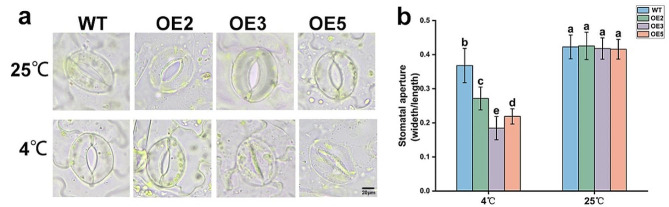
Observation of stomata after low-temperature treatment. (a) The scale is 20 μm. (b) Stomatal opening in OE and WT lines under low-temperature stress. Data are shown as mean ± SE of three independent experiments (*n* = 3, biological replicates), with different letters representing significant differences at different levels (***P*** **< 0.05**)

Ultrastructural observations of stomata and stomatal width-to-length ratio measurements were conducted on OE and WT tobacco plant leaves after 6 h of treatment at 4 °C and 25 °C (Fig. 5). At 25 °C, little difference in stomatal openings was observed between OE and WT leaves. At 4 °C, WT’s stomatal opening decreased by 13%, while those of OE2, OE3, and OE5 decreased by 36.2%, 55.9%, and 47.3%, respectively. Thus, the stomatal openings of the OE lines were significantly reduced compared to those of WT.

### Analysis of flowering time of tobacco overexpressing ***PavHIPP16*** gene and expression of tobacco flowering-related genes

**Fig. 6:**
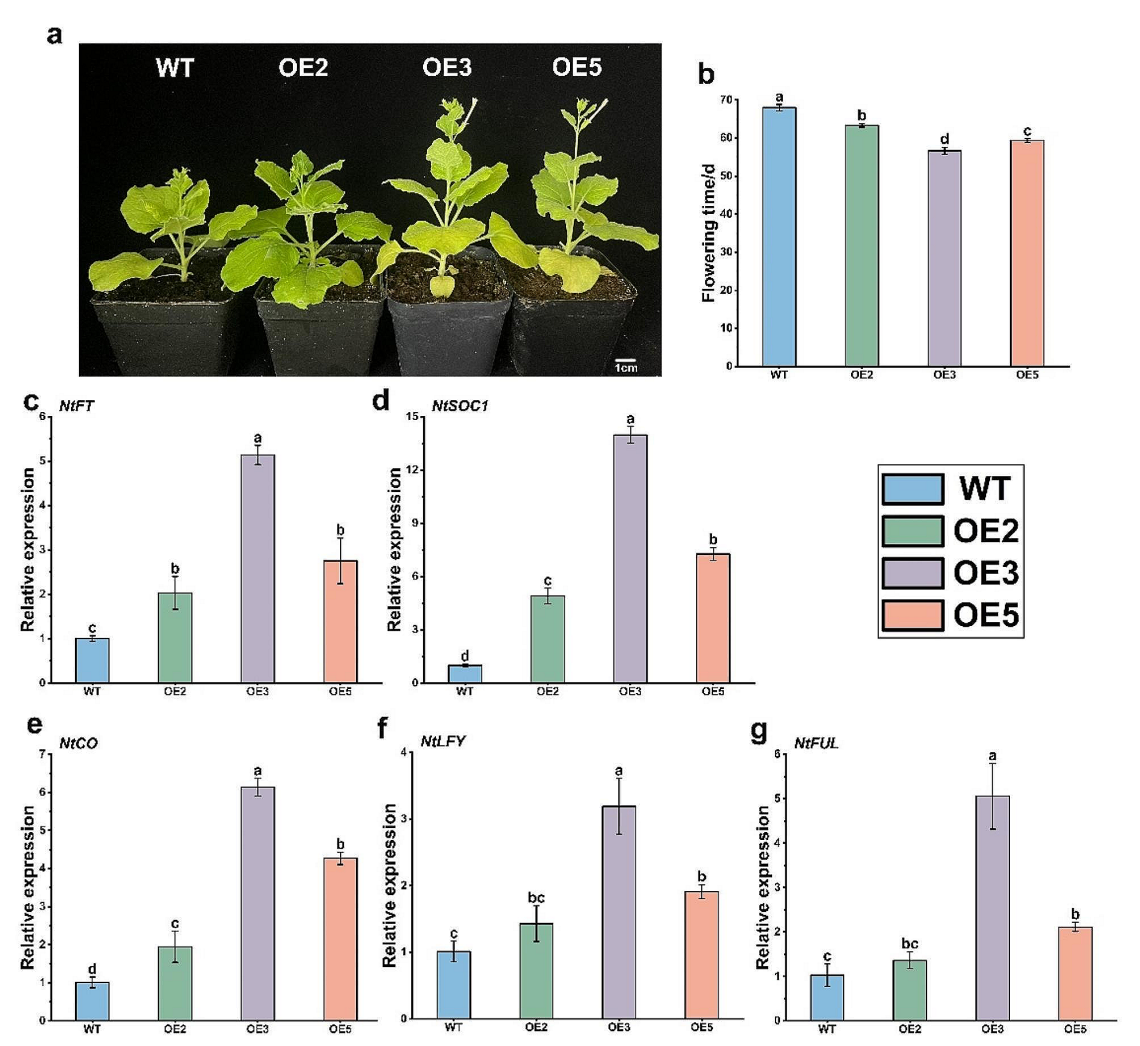
Phenotypic observation and related gene expression analysis of OE and WT. (**a**) WT and OE lines tobacco flowering; (**b**) WT and OE tobacco flowering time statistics; (**c-g**) represent the expression levels of *NtFT*, *NtSCO1*, *NtCO*, *NtLFY*, and *NtFUL* respectively. Data are shown as mean ± SE of three independent experiments (*n* = 3, biological replicates), with different letters representing significant differences at different levels (***P*** **< 0.05**)

After *PavHIPP16* heterologous transformation, phenotypic statistics analysis was conducted on each OE and WT lines. According to Fig. 6a-b, OE3 exhibited the earliest flowering time among the OE lines, while OE2 displayed the latest flowering time. However, all three OE lines flowered earlier and showed significant differences from WT.

*PavHIPP16* overexpression in plants resulted in a conspicuous early flowering phenotype, suggesting that the *PavHIPP16* upregulation in transgenic tobacco may induce changes in downstream gene expression. To validate this hypothesis, qRT-PCR was performed to detect the expression levels of these genes (Fig. 6c-g). The results revealed that OE showed a significant increase in the expression of tobacco flowering promoters compared to the WT. In particular, *flowering locus T* (*FT*), *CONSTANS* (*CO*), *suppressor of constans 1* (*SOC1*), *leafy* (*LFY*), and *fruitful* (*FUL*) exhibited significantly higher expression levels than those observed in WT. These findings suggest that these genes may be the downstream regulators of *HIPP16*-induced flowering and that *PavHIPP16* overexpression leads to elevated expression of downstream factors involved in flowering promotion.

### Changes in physiological and biochemical indexes of tobacco overexpressing ***PavHIPP16*** under low-temperature stress

**Fig. 7:**
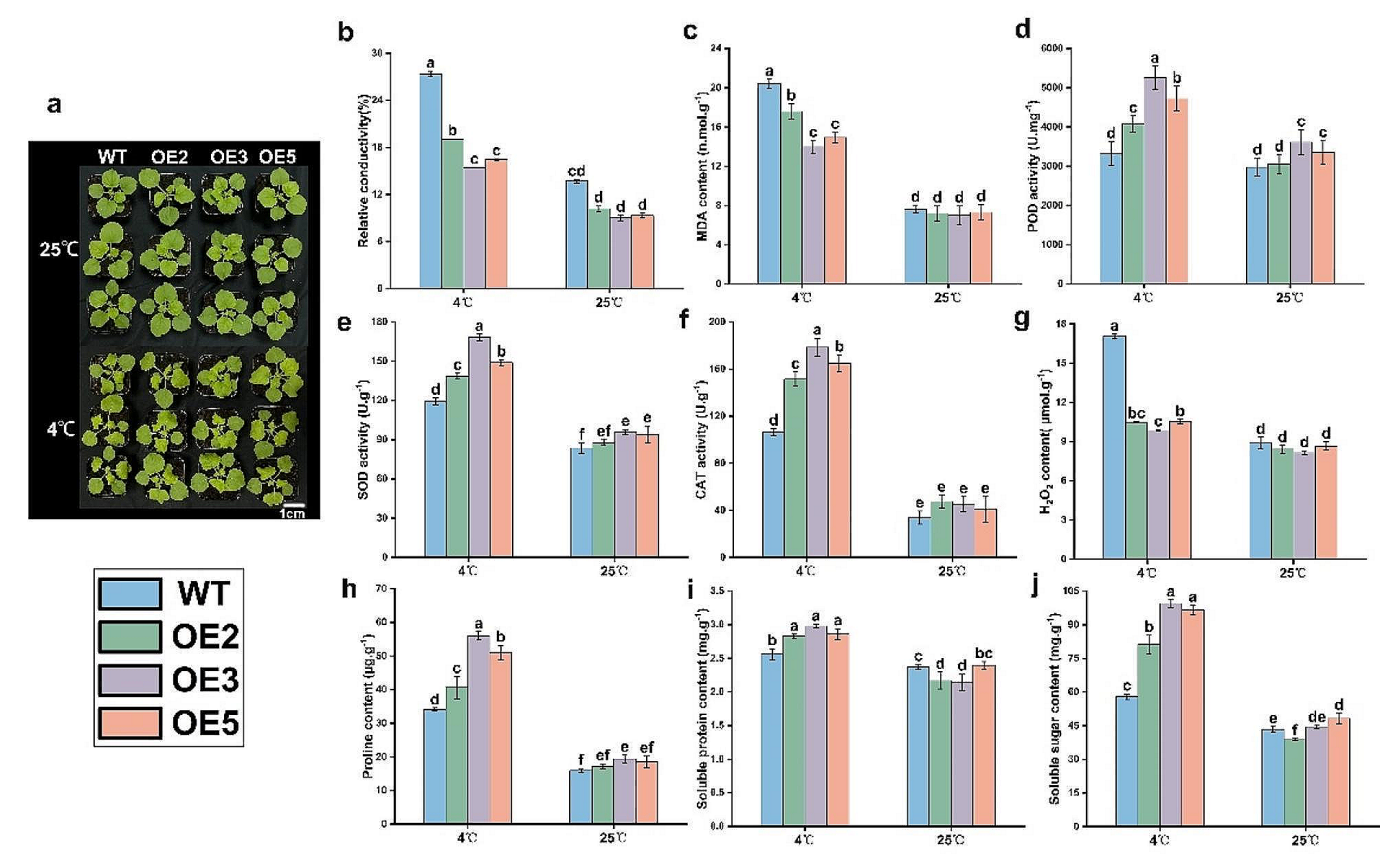
Enzyme activity tests after low-temperature treatment. (**a**) 4 °C treatment of OE and WT tobacco for 7 d; (**b-j**) Represent relative conductivity, MDA content, POD activity, SOD activity, CAT activity, H_2_O_2_ content, proline content, soluble protein content, and soluble sugar content respectively. Data are shown as mean ± SE of three independent experiments (*n* = 3, biological replicates), with different letters representing significant differences at different levels (***P*** **< 0.05**)

WT and OE (OE2, OE3, OE5) tobacco lines were subjected to treatment at 4 °C (Fig. 7a), and their leaves were collected to determine their physiological and biochemical indices under low-temperature stress conditions. Adverse conditions often lead to plant cell membrane rupture, resulting in cytoplasmic leakage and increased relative conductivity. Additionally, elevated MDA production exacerbates cell membrane damage, indicating a weaker protective capacity of plant organs and tissues with higher MDA content, thereby intensifying plant injury. The results demonstrated little difference in relative conductivity and MDA content between OE and WT tobacco at 25 °C (Fig. 7b-c). However, after low-temperature exposure at 4 °C, both OE and WT exhibited increased relative conductivity and MDA content; but those of OE were significantly lower than those of WT. Therefore, *PavHIPP16* overexpression reduced the lipid peroxidation of cell membranes, decreased the leaf cell membrane damage under low-temperature stress, and improved the tobacco’s adaptability to low temperatures compared with WT.

The POD enzyme scavenges excess free radicals in the plants, thereby enhancing plant resistance. SOD and POD work synergistically to effectively prevent plant injury caused by superoxide anion radicals. CAT is a crucial protective enzyme against ROS damage during plant stress, primarily responsible for eliminating the H_2_O_2_ produced in the plant. As shown in Fig. 7d-f, there is little difference in POD, SOD and CAT activities between OE and WT at 25 °C. However, the activities of antioxidant enzymes increased in the OE lines and were significantly higher than those of the WT lines after low-temperature treatment. The H_2_O_2_ contents of OE and WT elevated during low-temperature stress (Fig. 7g), and that of WT was significantly higher than that of OE. Therefore, *PavHIPP16* overexpression could reduce tobacco leaf damage by alleviating the effects of superoxide anion radicals and ROS.

The accumulation of proline, soluble protein, and soluble sugar serves as crucial osmoregulatory mechanisms in plants, particularly under adversity stresses. Soluble proteins exhibit strong hydrophilicity, and their contents can serve as a reliable indicator of cellular water retention capacity. Soluble sugars, including glucose, alginate, and sucrose, play a significant role in plant osmoregulation. To assess the osmoregulatory substance content of OE and WT, the contents of relevant osmoregulatory substances were examined (Fig. 7h-j). No substantial difference was observed in the proline, soluble protein, and soluble sugar contents among all plants at 25 °C. However, the contents of these osmoregulatory substances increased significantly following low-temperature treatment at 4 °C, with OE exhibiting notably higher contents compared to WT counterparts. Thus, when exposed to low-temperature stress, OE accumulated osmoregulatory substances more rapidly than WT, thus mitigating low-temperature damage to cell membranes and enhancing cold tolerance.

### Expression of cold stress-related genes in tobacco overexpressing ***PavHIPP16*** under low-temperature stress

**Fig. 8:**
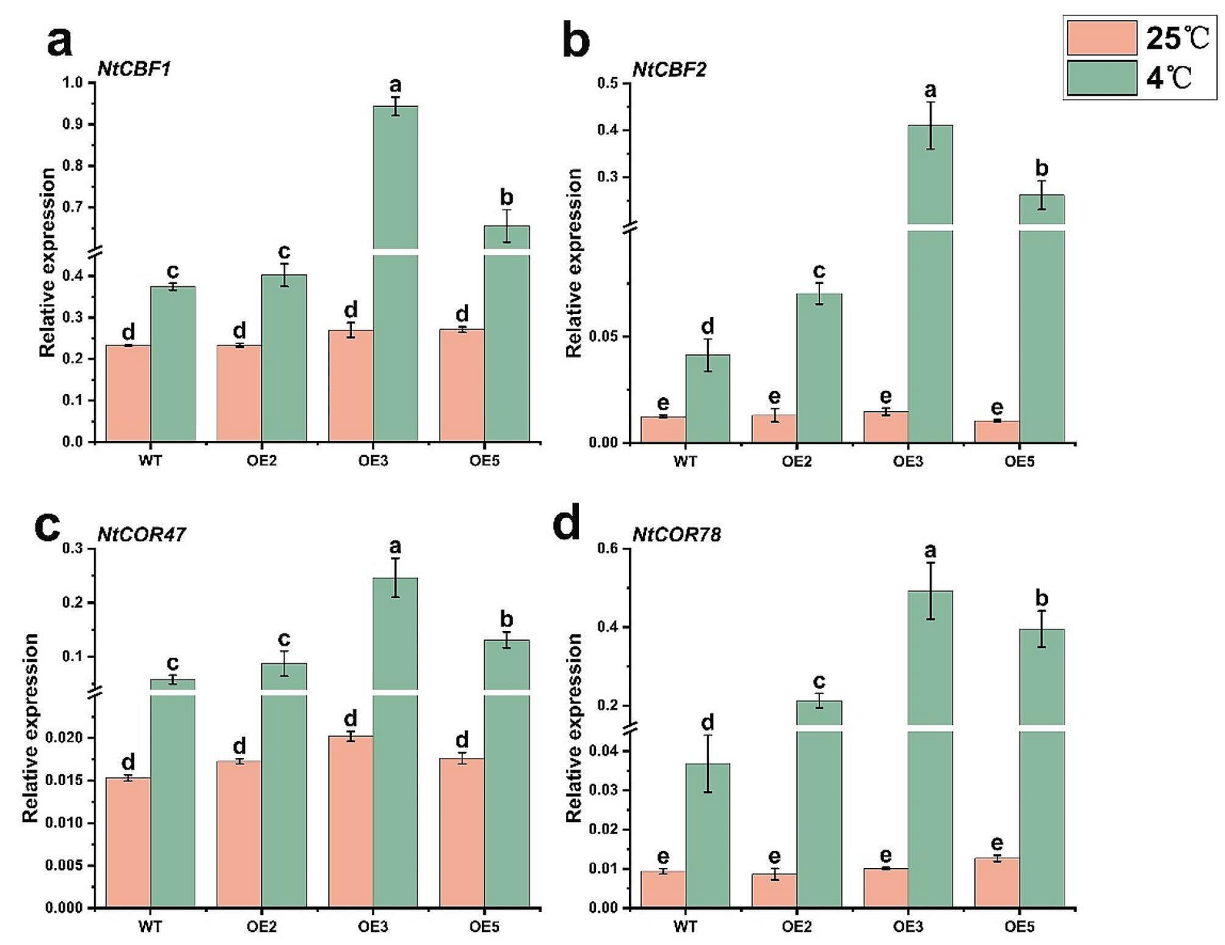
Expression analysis of stress-responsive genes in different OE and WT under low-temperature stress and control conditions. (**a-d**) represent *NtCBF1*, *NtCBF2*, *NtCOR47* and *NtCOR78* respectively. Note: WT: Wild tobacco line; OE2, OE3, OE5: Transgenic tobacco lines. Data are shown as mean ± SE of three independent experiments (*n* = 3, biological replicates), with different letters representing significant differences at different levels (***P*** **< 0.05**)

The expression of cold-regulated genes in plants is influenced by low-temperature stress, thereby impacting cellular metabolism, growth, and development. To investigate the effect of *PavHIPP16* on cold stress signaling through a CBF-dependent pathway, OE and WT lines were subjected to low-temperature treatment. The expression levels of CBF-COR pathway-related genes (*NtCBF1*, *NtCBF2*, *NtCOR47*, and *NtCOR78*) in tobacco were examined in different tobacco lines. (Fig. 8). Under normal conditions at 25 °C, there was no significant difference in the expression levels of these genes among all lines. However, the expression of all these genes after exposure to low-temperature stress at 4 °C, was up-regulated, and those in OE were significantly higher compared with WT. These findings suggest that *PavHIPP16* can enhance plant tolerance to low-temperature stress by modulating the expression of CBF and its downstream target genes.

### Analysis of ***PavHIPP16*** and ***PavbHLH106*** protein interactions

**Fig. 9:**
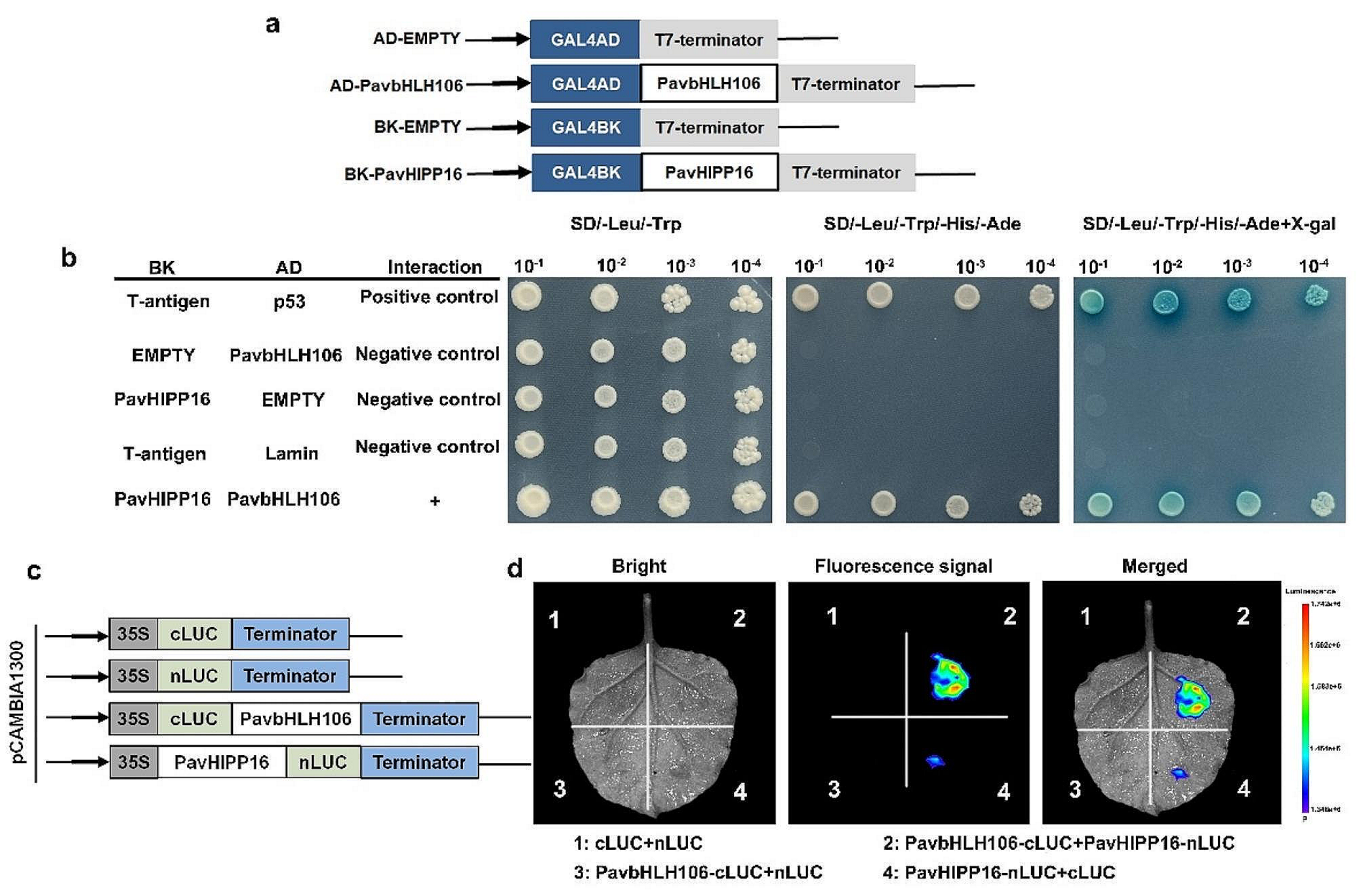
Heterodimerization of PavbHLH106 and PavHIPP16. (**a**) Schematic diagram of the vector construction.; (**b**) The results of the Y2H experiments indicate that PavbHLH106 and PavHIPP16 interact. Yeast containing AD and BD recombinant plasmids grow and turn blue in SD/-Trp/-Leu/-Ade/-His medium. (**c**) Schematic diagram of the vector construction. (**d**) The results of luciferase complementation experiments indicate that PavbHLH106 and PavHIPP16 interact. The injected regions containing nLUC and cLUC recombinant plasmids were luminescent and brighter than other controls

The regulatory network of PavbHLH106 and PavHIPP16 interaction was further investigated through Y2H. The results showed that all yeast combinations could grow normally on SD/-Leu/-Trp medium, indicating successful expression in the yeast (Fig. 9b). Notably, yeast strains co-expressing pGADT7-PavbHLH106 and pGBKT7-PavHIPP16 grew normally on SD/-Ade/-His/-Leu/-Trp medium and caused the reporter gene expression in the yeast to make the X-Gal colorimetric substrate blue, similar to the growth status of the positive control. In contrast, the control failed to grow in SD/-Ade/-His/-Leu/-Trp.

In the luciferase complementation assay, fluoro kinase is enzymatically cleaved into two functional fragments, namely the C-terminal and N-terminal fragments (cLUC and nLUC), which interact with each other. In this assay, PavbHLH106 and PavHIPP16 were fused to cLUC and nLUC, respectively. If these two proteins can interact with each other, the cLUC and nLUC fragments of fluoro kinase will be nearby within a specific region, allowing for their correct assembly into fusion proteins. This enables the realization of fluorophore enzyme activity and subsequent fluorescence detection. A mixture of PavbHLH106-cLUC + PavHIPP16-nLUC, PavbHLH106-cLUC + nLUC, PavHIPP16-nLUC + cLUC, and cLUC + nLUC were injected into four different regions of tobacco leaves to measure LUC activity. The results showed (Fig. 9d) that significant LUC activity could be detected in the region co-injected with PavbHLH106-cLUC and PavHIPP16-nLUC. Conversely, no LUC activity was observed in the remaining injected regions, indicating that interaction between PavbHLH106 and PavHIPP16 proteins occurred exclusively within this region.

## Discussion

Plant growth and development are affected by various environmental factors, including biotic and abiotic factors, with a low temperature being one of the most important. Plant response to low temperature begins with protein receptors on the plasma membrane, including receptor kinases, membrane kinases, and ion channels, which may be involved in the response mechanism to low-temperature stress [25, 26]. Some other proteins, such as link HIPPs, may respond to cold stress through these mechanisms. HIPPs can affect plant development, growth, and abiotic stresses, and isoprenylation of their specialized structural proteins is commonly associated with plant stress responses [27, 28]. Therefore, it is important to study the action mechanism of HIPPs on low-temperature stress.

This study conducted a functional characterization of sweet cherry *PavHIPP16* in tobacco and validated the interaction with a basic helix-loop-helix (bHLH) protein, suggesting a potential role in their resistance to cold stress. PavHIPP16 is more closely related to the HIPPs of Rosaceae, which are conserved during evolution. Various environmental adaptations have accumulated during plant evolution, accompanied by the evolution of genes to form a more complex regulatory network to cope with various environmental challenges [29].

### Multiple biological functions of HIPPs

Seed germination is regulated by many external and internal factors [30], and ABA and GAs are the most important phytohormones that regulate germination [31–33]. Phytohormones can regulate seed germination by affecting the activity of hydrolytic enzymes or by regulating the accumulation of non-enzymatic factors such as ROS [34, 35]. This study found that the germination rate of OE tobacco seeds was significantly higher than that of WT (Fig. 3), suggesting that *PavHIPP16* overexpression promoted seed germination. The root length and fresh weight of OE were significantly higher than those of WT (Fig. 4). Therefore, it was hypothesized that *PavHIPP16* overexpression increased the growth capacity of tobacco seedlings and that the seedling growth capacity of OE was also significantly higher than that of WT at low temperatures.

Stomata are important gas and water exchange channels between leaves and the external environment and are essential for photosynthesis and transpiration in plants. When plants are subjected to environmental stress, a large number of physiological responses are induced in plants, including stomatal closure [36]. Stomata respond in various ways to mitigate the stress-induced damage, thereby increasing plant resilience. Under low-temperature conditions, the stomata close, and the net photosynthetic rate decreases, thereby reducing plant damage [37]. The stomatal width-to-length ratio and stomatal opening of tobacco were significantly reduced after low-temperature stress, and the stomatal opening of OE was significantly smaller than that of WT (Fig. 5). Thus, *PavHIPP16* overexpression promotes the stomatal closure of tobacco, which improves its cold hardiness.

Flowering is crucial for plant growth and development and is intricately regulated by various factors such as plant photoperiod, temperature, and hormones. These factors work synergistically to orchestrate the complex plant flowering [38]. As regulatory genes, bHLH transcription factors are widely involved in various plant metabolic developmental processes, including flowering and fruit development [39]. The *FT* is a major component of tobacco fluorogenic hormones and, like *SOC1*, acts as a signal-integrating factor that interacts with the floral meristem genes *LFY* and *FUL* to induce flowering. CO protein is a plant-specific transcriptional activator that affects flowering time by directly regulating *FT* expression. This study found that *PavHIPP16* overexpression resulted in earlier flowering of transgenic tobacco lines than WT (Fig. 6), and the expression of tobacco flowering-related regulatory genes was significantly increased, possibly implying that *PavHIPP16* could accelerate flowering time by promoting *NtFT*, *NtSCO1*, *NtCO*, *NtLFY*, and *NtFUL* expression.

By affecting enzyme activity and cell membrane function, low temperatures often lead to cell dehydration, affecting cell metabolism and destabilizing autophagy [40, 41]. Plant damage caused by low-temperature stress is severe and often irreversible, commencing with structural damage to the cell membrane, i.e., water loss and osmotic potential changes, such as changes in relative conductivity and MDA content. These changes increase cell membrane permeability and affect the normal plant physiological processes [42]. Signaling is an adaptive regulatory mechanism for plants to cope with stresses. Under low-temperature stress, the cytoprotective enzymes POD, SOD, and CAT, which are part of the ROS scavenging system, elevate to scavenge the accumulated excess ROS and to improve plant tolerance [43, 44]. Cell membrane stability preservation is essential for the cold tolerance of plant under low-temperature stress, which disrupts the balance in osmoregulatory substances such as proline, soluble protein, and soluble sugar that help to cope with various biotic and abiotic stresses [45]. In the present study, antioxidant enzymes (POD, SOD, CAT) activities and osmoregulatory substances (proline, soluble proteins and soluble sugars) were significantly higher in the OE line than in WT, and the relative conductivity, MDA content and H_2_O_2_ content were significantly lower in OE than in WT. Therefore, higher POD, SOD and CAT activities in OE enhanced the ability of tobacco to scavenge H_2_O_2_ and decreased the and reduced the damage induced by low temperature, thus improving the cold tolerance of tobacco.

Plants resist low temperature by regulating membrane system components and osmotically regulated substances, which promotes the expression of cold stress-responsive genes and activates the ROS scavenging system [46, 47]. Relative conductivity reflects the degree of damage to the cell membrane damage, and a large number of electrolytes are extravasated from the cell with disrupted membranes, thus increasing the conductivity in solution [48]. Maintaining cell membrane integrity and stability under low-temperature stress is essential for plant cold resistance. Under low temperature, membrane lipid peroxidation affects the structure and function of plant cell membranes, thus increasing cell membrane permeability and ROS accumulation. The MDA content can reflect the degree of membrane lipid peroxidation and cellular damage [49]. Therefore, WT sustained greater cell membrane damage, while OE showed reduced membrane lipid peroxidation and increased stress tolerance.

Plant under low-temperature stress maintain cellular water by accumulating osmotic solutes, thereby regulating osmotic potential and pressure. Meanwhile, they reduce cellular osmotic potential and increase water uptake by increasing proline content, which promotes protein hydration, soluble protein content, and soluble sugar content, thus initiating plant defense mechanisms [50, 51]. Based on proline, soluble protein, and soluble sugar contents (Fig. 7h-j), OE lines were found to be less damaged at low temperature.

### Interaction of HIPPs with other proteins

A key plant response to low temperature is transducing the previously perceived signals and activating the related transcription factors to regulate the expression of low-temperature-related genes, thus gradually adapting to the low temperature. Among them, the perceived signals will activate the C-repeat/dehydration-responsive element binding factor (CBF) pathway to resist low-temperature stress, namely, the CBF-dependent pathway [52, 53]. The other pathway is called the non-CBF-dependent pathway. A large number of transcription factors associated with low-temperature response have been identified in tobacco that can regulate downstream CBF pathways, such as CBF1/2/3, ICE1, COR47, and bHLH [54, 55]. *NtbHLH123* is a transcription factor that regulates ROS scavenging-associated genes and stress-responsive genes by binding to the G-box/E-box motif in the promoter of *NtCBF*, thereby improving cold tolerance in tobacco [56].

Plants resist low temperature also by regulating the expression of multiple functional genes, and these mechanisms are accomplished through the different functions of various genes. Hence, studying the response process of these resistance genes and their regulatory networks is of great significance to understanding plant cold resistance. The ability of HIPP family members to respond to low-temperature stress has been reported in wheat [12], rice [9], and tomato [57]. In grapes, *VvHIPP21* can respond to low temperatures with HOS1 through the ubiquitination and degradation of ICE1 negative regulation. *VvHIPP21* overexpression significantly represses *CBF1*, *-2*, *-3*, and CBF-regulated genes (*COR*) in a CBF pathway-dependent manner [58]. The transcriptional cascade is a key regulatory framework of the low-temperature signaling pathway mediated by CBF regulators.

HIPP proteins can interact with other proteins to participate in plant stress tolerance. For instance, *HIPP26* from *Arabidopsis* interacts with a zinc-finger protein transcription factor, ATHB29, when subjected to low-temperature stress, and *HIPP26* mutation suppresses the expression of relevant stress-responsive genes regulated by ATHB29 [8]. The bHLH transcription factors are involved in plant growth and developmental processes, including flowering and fruit development [39]. For example, the *Arabidopsis* bHLH protein family FLOWERING BHLH 1 (FBH1), FBH2, FBH3, and FBH4 bind to the E-box *cis*-acting element of the CO promoter to regulate *CO* gene expression, and *AtFBH* overexpression promotes the up-regulation of the expression of *AtCO* expression, leading to early flowering in *Arabidopsis* [59]. CIB1, CIB2, CIB4, and CIB5 proteins work together to regulate flowering initiation, and they can directly bind to the *FT* promoter to promote FT transcription in plants [60]. PavbHLH28 can directly bind to the *POD2* gene promoter and induce its gene expression, and *PavbHLH28* overexpression enhances cold tolerance in plants [61]. Efforts have been made to screen proteins interacting with PavbHLH106 by Y2H [24], yielding PavHIPP16. This study verified the protein interactions by Y2H and luciferase complementation assays, and previous studies have demonstrated PavbHLH106’s involvement in low-temperature stress response, with elevated expression at low temperature [62]. The results of this study showed that *PavHIPP16* overexpression significantly increased the expression of *NtCBF1*, *NtCBF2*, *NtCOR47*, and *NtCOR78* under low-temperature stress (Fig. 8). *PavHIPP16* overexpression in transgenic plants may enhance cold tolerance in tobacco through the CBF-COR pathway. Therefore, it is hypothesized that the *HIPP* genes can induce the expression of downstream CBF-COR pathway-related genes, thereby affecting the cold tolerance of plants. This finding provides a basis for resolving the CBF-COR signaling pathway.

## Conclusion

Tobacco with *PavHIPP16* overexpression showed a significant reduction in relative conductivity and MDA content after low-temperature stress. Specifically, the activity of the antioxidant enzymes CAT, SOD, and POD were significantly elevated, the H_2_O_2_ content was significantly reduced, and the proline, soluble protein, and soluble sugar contents were significantly increased. *PavHIPP16* overexpression not only affected the expression of low-temperature-related genes but also induced the expression of flowering-critical genes. In addition, Y2H and luciferase complementation experiments confirmed the protein interactions between PavHIPP16 and PavbHLH106. Thus, this study reveals for the first time that *PavHIPP16* is essential for enhancing plant cold tolerance under low-temperature stress.

## Materials and methods

### Plant materials

The sweet cherry cultivar ‘Cordia’ was used in this research, sourced from the Deciduous Fruit Tree Industry-University-Research Base of the Key Laboratory of Mountain Plant Resource Conservation and Germplasm Innovation of the Ministry of Education, Guizhou University. The genetic transformation experiment employed *Nicotiana benthamiana* as the material, with seeds preserved at the Key Laboratory of Mountain Plant Resource Conservation and Germplasm Innovation of the Ministry of Education, Guizhou University.

### Plant gDNA extraction and plant cDNA synthesis

Plant gDNA and total RNA were extracted using the Plant DNA Extraction Kit (Tengen, China) and the Total RNA Extraction Kit for Polysaccharide and Polyphenol Plants (Omega, USA), respectively, with minor modifications as described in the manual. The concentration and quality of the extracted RNA and DNA were determined using an enzyme labeling instrument, Multiskan GO (Thermo Fisher, USA). At the same time, the stability of the RNA was assessed through 1% agarose gel electrophoresis containing the nucleic acid dye GoldView (10,000×). The extracted RNA served as a template for cDNA synthesis using the Revere Transcription Kit (Genstar, China), following the provided instructions.

### Genetic transformation of tobacco by leaf disk method

The complete CDS sequence of *PavHIPP16* was retrieved from the genetic database of Rosaceae database (https://www.rosaceae.org/). Subsequently, the *PavHIPP16* was cloned with sweet cherry leaf cDNA, and inserted into the pCambia1301-35s vector using the seamless cloning technique to generate a recombinant plasmid pCambia1301-35s-*PavHIPP16*. The primers used in this study are provided in the Supplementary Table.

The WT tobacco seeds were sterilized under aseptic conditions. Specifically, they were initially sterilized with 75% alcohol for 30 s and then rinsed 2 to 3 times with sterile water. Then, they were soaked in 10% sodium hypochlorite for 10 min and washed 2 to 3 times again with sterile water. The seeds were inoculated into a solid medium. Next, 1-month-old tobacco leaves were selected as the transformed materials, and the leaf disc method was used [63]. All experiments were conducted under aseptic conditions [64]. The leaves were collected for gDNA extraction to detect positive lines. *Nt-Actin* was used as an internal reference gene in tobacco, and different lines’ expression levels were assessed. OE *PavHIPP16* transgenic tobacco lines were selected for subsequent studies.

### Observation of germination rate and root length at low temperature

After obtaining the OE lines, T_3_ generation tobacco was obtained through continuous PCR identification and resistance screening for subsequent research. Seeds and seedlings from T_3_ generation OE lines (OE2, OE3, OE5) were specifically selected for low-temperature stress treatment to observe and compare phenotypic differences between WT and OE.

Seeds of OE and WT lines, 25 each, were sown in the medium, which was transferred into a plant growth chamber for 10 d to observe and record the daily germination. Seedlings of relatively uniform size and growth were selected and transplanted into a new medium for vertical culture at different temperatures (4 °C, 8 °C, and 25 °C). The root length and root fresh weight were determined after 10 d. Three biological replicates were used for each treatment.

### Determination of physiological traits under low-temperature treatment

Tobacco was collected from the low-temperature treatment (4℃) and control groups after 6 h. Then, the epidermis was torn off along the leaf veins on the abaxial surface of similarly sized leaves, which were prepared and photographed for observation with a biomicroscope. For each line, the transverse and longitudinal diameters of 50 stomata were counted, and the stoma openness was determined by the ratio of transverse/longitudinal diameters, which was plotted into a bar chart for analysis.

The tobacco seeds, both OE and WT, were sown and allowed to grow for approximately 2 weeks and then transplanted into an artificial climate chamber (16 h/8 h, 23 ± 2 °C) for 3 weeks. The OE and WT lines were treated at 4 °C for 7 d, while the control was maintained at 25 °C under normal conditions. After stress induction, tobacco leaves were collected, carefully packaged in tin foil, and immediately snap-frozen in liquid nitrogen at -80 °C before storage. These frozen leaves can be used as templates for subsequent total RNA extraction to detect resistance gene expression levels and measure physiological and biochemical indices by a qRT-PCR instrument (Analytik Jena, Germany).

The relative conductivity y was determined using a Jenco 3020 conductivity meter (JENCO, China) and the corresponding method [65]. Finally, relative conductivity was calculated using the formula: relative conductivity (%) = C1/C2 × 100%, where C1 represents the conductivity before boiling, C2 represents the conductivity after boiling, and the unit of conductivity is us.cm^− 1^. The POD, SOD, and CAT, as well as proline, soluble protein, soluble sugar, H_2_O_2_, and MDA were determined using the kit provided by Solarbio Biotechnology Co. Ltd. (Solarbio, China) and analyzed with a UV spectrophotometer (PHILES, China).

### Expression analysis of low-temperature-related genes and flowering-related genes

The expression levels of stress-related genes in OE tobacco under low-temperature stress were further investigated using the resistance-related genes *NtCBF1*, *NtCBF2*, *NtCOR47*, and *NtCOR78*, and flowering-related genes *NtFT*, *NtSCO1*, *NtCO*, *NtLFY*, and *NtFUL*. The primer sequences can be found in the Supplemental Table. Tobacco *Nt-Actin* was selected as an internal reference gene, and the gene expression levels were analyzed by qRT-PCR. Three biological replicates and three technical replicates were performed for each strain.

### Y2H and luciferase complementation assay

The interaction of PavbHLH106 with PavHIPP16 was further investigated via Y2H and luciferase complementary assay techniques [66]. The CDS of PavbHLH106 and PavHIPP16 were cloned into pGADT7 and pGBKT7 vectors, respectively, and then cotransfected into Y2H Gold yeast. Positive transformants were screened to verify their interactions by culturing them on SD/-Trp/-Leu medium and SD/-Ade/-Trp/-Leu/-His medium. Additionally, PavbHLH106-cLUC and PavHIPP16-nLUC vectors were constructed. *Agrobacterium* strain GV3101 was transformed and co-injected into *N. benthamiana*. The fluorescence intensity was measured using a plant live imaging system (PlantView600, China). The primers used are listed in the Supplemental Table.

### Statistical analysis of data

The relative expression of all relevant genes (*PavHIPP16*, *NtCBF1*, *NtCBF2*, *NtCOR47*, *NtCOR78*, *NtFT*, *NtSCO1*, *NtCO*, *NtLFY*, and *NtFUL*) were calculated using the 2^−ΔCT^ method [67]. Statistical significance was assessed using SPSS 21.0 software, with Duncan’s test applied and a significance level set at *P* < 0.05. Bar graphs were generated using Origin 2022.

### Electronic supplementary material

Below is the link to the electronic supplementary material.

Supplementary Material 1

Supplementary Material 2

Supplementary Material 3: An additional movie file shows this in more detail [see Additional file 1: Supplemental Table]

## Data Availability

All data generated or analyzed during this study are included in this published article [and its supplementary information file].

## Abbreviations

ABA: Abscisic acid
bHLH: Basic helix-loop-helix
CAT: Catalase
CBF: C repeat binding factor
CDS: Coding sequence
CO: CONSTANS
COR: Cold-regulated
DNA: Deoxyribonucleic acid
FT: Flowering locus T
FUL: Fruitful
GAs: Gibberellins
H_2_O_2_: Hydrogen peroxide
HIPP: Heavy metal-associated isoprenylated plant proteins
LFY: Leafy
LUC: Luciferase
MDA: Malonaldehyde
OE: Overexpression
ORF: Open reading frame
PCR: Polymerase chain reaction
POD: Peroxidase
qRT-PCR: Quantitative real-time polymerase chain
RNA: Ribonucleic acid
ROS: Reactive oxygen species
SOC1: Suppressor of constans 1
SOD: Superoxide dismutase
WT: Wild type
Y2H: Yeast two-hybrid assay

## References

[1] Zhang H, Zhang X, Liu J, Niu Y, Chen Y, Hao Y et al. Characterization of the heavy-metal-associated isoprenylated plant protein (HIPP) gene family from triticeae species. Int J Mol Sci. 2020;21. 10.3390/ijms21176191 PMC7504674 32867204

[2] Bull PC, Cox DW (1994). Wilson disease and Menkes disease: new handles on heavy-metal transport. Trends Genet.

[3] Barth O, Vogt S, Uhlemann R, Zschiesche W, Humbeck K (2009). Stress induced and nuclear localized HIPP26 from Arabidopsis thaliana interacts via its heavy metal associated domain with the drought stress related zinc finger transcription factor ATHB29. Plant Mol Biol.

[4] Tehseen M, Cairns N, Sherson S, Cobbett CS (2010). Metallochaperone-like genes in Arabidopsis thaliana. Metallomics.

[5] Arnesano F, Banci L, Bertini I, Ciofi-baffoni S, Molteni E, Huffman DL (2002). Metallochaperones and metal-transporting ATPases: a comparative analysis of sequences and structures. Genome Res.

[6] Furukawa Y, Lim C, Tosha T, Yoshida K, Hagai T, Akiyama S et al. Identification of a novel zinc-binding protein, C1orf123, as an interactor with a heavy metal-associated domain. PLoS ONE. 2018;13. 10.1371/journal.pone.0204355 PMC6160046 30260988

[7] Gao W, Xiao S, Li HY, Tsao SW, Chye ML (2009). *Arabidopsis thaliana* acyl-CoA-binding protein ACBP2 interacts with heavy-metal-binding farnesylated protein AtFP6. New Phytol.

[8] Suzuki N, Yamaguchi Y, Koizumi N, Sano H (2002). Functional characterization of a heavy metal binding protein CdI19 from Arabidopsis. Plant Journal: Cell Mol Biology.

[9] Jeon J, Kim J (2013). Cold stress signaling networks in *Arabidopsis*. J Plant Biology.

[10] Zhao YN, Wang MQ, Li C, Cao HW, Rono JK, Yang ZM. The metallochaperone *OsHIPP56* gene is required for cadmium detoxification in rice crops. Environ Exp Bot. 2022;193.

[11] Zhang X, Feng H, Feng C, Xu H, Huang X, Wang Q (2015). Isolation and characterisation of cDNA encoding a wheat heavy metal-associated isoprenylated protein involved in stress responses. Plant Biol.

[12] Zschiesche W, Barth O, Daniel K, Boehme S, Rausche J, Humbeck K (2015). The zinc-binding nuclear protein HIPP3 acts as an upstream regulator of the salicylate-dependent plant immunity pathway and of flowering time in *Arabidopsis thaliana*. New Phytol.

[13] Tran LSP, Nakashima K, Sakuma Y, Osakabe Y, Qin F, Simpson SD (2007). Co-expression of the stress-inducible zinc finger homeodomain ZFHD1 and NAC transcription factors enhances the expression of the ERD1 gene in *Arabidopsis*. Plant Journal: Cell Mol Biology.

[14] Chen G, Xiong S (2021). OsHIPP24 is a copper metallochaperone which affects Rice Growth. J Plant Biology.

[15] Guo T, Weber H, Niemann MCE, Theisl L, Leonte G, Novak O (2021). *Arabidopsis* HIPP proteins regulate endoplasmic reticulum-associated degradation of CIOX proteins and cytokinin responses. Mol Plant.

[16] Tan DX, Manchester LC, Di MP, Martinez GR, Prado FM (2007). Reiter RJ. Novel rhythms of N1-acetyl-N2-formyl-5-methoxykynuramine and its precursor melatonin in water hyacinth: importance for phytoremediation. Faseb J.

[17] Rugienius R, Šnipaitiene L, STanienė G, Šikšnianienė JB, Haimi P, Baniulis D (2016). Cold acclimation efficiency of different Prunus and Fragaria species and cultivars in vitro[J]. Zemdirbyste.

[18] Gilmour SJ, Fowler SG, Thomashow MF (2004). Arabidopsis transcriptional activators CBF1, CBF2, and CBF3 have matching functional activities [J]. Plant Mol Biol.

[19] Thomashow MF (2010). Molecular basis of Plant Cold Acclimation: insights gained from studying the CBF Cold Response Pathway. Plant Physiol.

[20] Song J, Guo B, Song F, Peng H, Yao Y, Zhang Y (2011). Genome-wide identification of gibberellins metabolic enzyme genes and expression profiling analysis during seed germination in maize [J]. Gene.

[21] Casson SA, Hetherington AM (2014). Phytochrome B is required for light-mediated systemic control of Stomatal Development [J]. Curr Biol.

[22] Turan MA, Elkarim AHA, Taban N, Taban S (2009). Effect of salt stress on growth, stomatal resistance, proline and chlorophyll concentrations on maize plant [J]. Afr J Agric Res.

[23] Van BF, Slooten L, Stassart JM, Moens T, Botterman J, Van MM (1999). Overproduction of *Arabidopsis thaliana* FeSOD confers oxidative stress tolerance to transgenic maize [J]. Plant Cell Physiol.

[24] Hou Q, Shen T, Yu R, Deng H, Wen X, Qiao G. Functional analysis of sweet cherry *PavbHLH106* in the regulation of cold stress [J]. Plant Cell Rep. 2024;43(1). 10.1007/s00299-023-03115-5 38133822

[25] Guo X, Liu D, Chong K (2018). Cold signaling in plants: insights into mechanisms and regulation [J]. J Integr Plant Biol.

[26] Shi Y, Ding Y, Yang S (2018). Molecular reculation of CBF Sicnalinc in Colc acclimation [J]. Trends Plant Sci.

[27] Rono JK, Sun D, Yang ZM, Metallochaperones. A critical regulator of metal homeostasis and beyond [J]. Gene. 2022;822. 10.1016/j.gene.2022.146352 35183685

[28] Hala M, Zarsky V. Protein prenylation in plant stress responses [J]. Molecules. 2019;24(21). 10.3390/molecules24213906 PMC6866125 31671559

[29] Feller A, Machemer K, Braun EL, Grotewold E (2011). Evolutionary and comparative analysis of MYB and bHLH plant transcription factors [J]. Plant J.

[30] Chen BX, Peng YX, Yang XQ, Liu J (2021). Delayed germination of *Brassica parachinensis* seeds by coumarin involves decreased GA_4_ production and a consequent reduction of ROS accumulation [J]. Seed Sci Res.

[31] Diaz-Vivancos P, Barba-Espin G, Antonio Hernandez J (2013). Elucidating hormonal/ROS networks during seed germination: insights and perspectives [J]. Plant Cell Rep.

[32] Shu K, Liu XD, Xie Q, He ZH (2016). Two faces of one seed: hormonal regulation of Dormancy and germination [J]. Mol Plant.

[33] Tuan PA, Kumar R, Rehal PK, Toora PK, Ayele BT. Molecular mechanisms underlying abscisic acid/gibberellin balance in the control of seed dormancy and germination in cereals [J]. Front Plant Sci. 2018;9. 10.3389/fpls.2018.00668 PMC5974119 29875780

[34] Ye N, Zhu G, Liu Y, Zhang A, Li Y, Liu R (2012). Ascorbic acid and reactive oxygen species are involved in the inhibition of seed germination by abscisic acid in rice seeds [J]. J Exp Bot.

[35] Bailly C (2019). The signaling role of ROS in the regulation of seed germination and dormancy [J]. Biochem J.

[36] Zhou R, Yu X, Ottosen CO, Zhao T (2020). High throughput sequencing of circRNAs in Tomato leaves responding to multiple stresses of Drought and Heat [J]. Hortic Plant J.

[37] Kollist H, Nuhkat M, Roelfsema MRG (2014). Closing gaps: linking elements that control stomatal movement [J]. New Phytol.

[38] Haberman A, Ackerman M, Crane O, Kelner JJ, Costes E, Samach A (2016). Different flowering response to various fruit loads in apple cultivars correlates with degree of transcript reaccumulation of a TFL1-encoding gene [J]. Plant J.

[39] Li H, Gao W, Xue C, Zhang Y, Liu Z, Zhang Y (2019). Genome-wide analysis of the bHLH gene family in Chinese jujube (*Ziziphus jujuba* Mill.) And wild jujube [J]. BMC Genomics.

[40] Guan Y, Hwarari D, Korboe HM, Ahmad B, Cao Y, Movahedi A et al. Low temperature stress-induced perception and molecular signaling pathways in plants [J]. Environ Exp Bot. 2023;207.

[41] Han J, Li X, Li W, Yang Q, Li Z, Cheng Z (2023). Isolation and preliminary functional analysis of FvICE1, involved in cold and drought tolerance in *Fragaria vesca* through overexpression and CRISPR/Cas9 technologies [J]. Plant Physiol Bioch.

[42] Long H, Li Z, Suo H, Ou L, Miao W, Deng W. Study on the mechanism of grafting to improve the tolerance of pepper to low temperature [J]. Agronomy-Basel. 2023;13(5).

[43] Luo P, Chen L, Chen Y, Shen Y, Cui Y. *RmZAT10*, a novel Cys2/His2 zinc finger transcription factor of *Rosa multiflora*, functions in cold tolerance through modulation of proline biosynthesis and ROS homeostasis [J]. Environ Exp Bot. 2022;198.

[44] Rezayian M, Niknam V, Ebrahimzadeh H (2020). Penconazole and calcium ameliorate drought stress in canola by upregulating the antioxidative enzymes [J]. Funct. Plant Biol.

[45] Per TS, Khan NA, Reddy PS, Masood A, Hasanuzzaman M, Khan MIR (2017). Approaches in modulating proline metabolism in plants for salt and drought stress tolerance: phytohormones, mineral nutrients and transgenics [J]. Plant Physiol Bioch.

[46] Jia Y, Ding Y, Shi Y, Zhang X, Gong Z, Yang S (2016). The *CBFs* triple mutants reveal the essential functions of *CBFs* in cold acclimation and allow the definition of CBF regulons in *Arabidopsis* [J]. New Phytol.

[47] Shi Y, Huang J, Sun T, Wang X, Zhu C, Ai Y, Gu H (2017). The precise regulation of different *COR* genes by individual CBF transcription factors in *Arabidopsis thaliana* [J]. J Integr Plant Biol.

[48] Baid V (1994). Low temperature and drought regulated gene expression in *bermudagrass* [J]. Turfgrass Environ Res Summ.

[49] Wang S, Liang D, Li C, Hao Y, Ma F, Shu H (2012). Influence of drought stress on the cellular ultrastructure and antioxidant system in leaves of drought-tolerant and drought-sensitive apple rootstocks [J]. Plant Physiol Bioch.

[50] Miller G, Suzuki N, Ciftci-Yilmaz S, Mittler R (2010). Reactive oxygen species homeostasis and signalling during drought and salinity stresses [J]. Plant Cell Environ.

[51] Fang Y, Xiong L (2015). General mechanisms of drought response and their application in drought resistance improvement in plants [J]. Cell Mol Life Sci.

[52] Stockinger E, Gilmour S, Thomashow M (1997). *Arabidopsis thaliana CBF1* encodes an AP2 domain-containing transcriptional activator that binds to the C-repeat/DRE, a cis-acting DNA regulatory element that stimulates transcription in response to low temperature and water deficit [J]. PNAS.

[53] Shi Y, Ding Y, Yang S (2015). Cold signal transduction and its interplay with phytohormones during cold acclimation [J]. Plant Cell Physiol.

[54] Chinnusamy V, Ohta M, Kanrar S, Lee B, Hong X, Agarwal M (2003). ICE1: a regulator of cold-induced transcriptome and freezing tolerance in *Arabidopsis*[J]. Genes Dev.

[55] Agarwal M, Hao Y, Kapoor A, Dong C, Fujii H, Zheng X (2006). A R2R3 type MYB transcription factor is involved in the cold regulation of CBF genes and in acquired freezing tolerance[J]. J Biol Chem.

[56] Zhao F, Li G, Hu P, Zhao X, Li L, Wei W et al. Identification of basic/helix-loop-helix transcription factors reveals candidate genes involved in anthocyanin biosynthesis from the strawberry white-flesh mutant [J]. Sci Rep. 2018;8. 10.1038/s41598-018-21136-z PMC5807450 29426907

[57] Manara A, Fasani E, Molesini B, Dalcorso G, Pennisi F, Pandolfini T et al. The tomato metallocarboxypeptidase inhibitor I, which interacts with a heavy metal-associated isoprenylated protein, is implicated in plant response to cadmium [J]. Molecules. 2020;25(3). 10.3390/molecules25030700 PMC7038206 32041288

[58] Zheng Q, Yu Q, Wu N, Yao W, Li J, Lv K et al. A grape VvHOS1-interacting HIPP protein (VvHIPP21) negatively regulates cold and drought stress [J]. Environ Exp Bot. 2023;207.

[59] Ito S, Song YH, Josephson-Day AR, Miller RJ, Breton G, Olmstead RG et al. FLOWERING BHLH transcriptional activators control expression of the photoperiodic flowering regulator *CONSTANS* in *Arabidopsis* [J]. PNAC. 2012;109(9): 3582-7. 10.1073/pnas.1118876109 PMC3295255 22334645

[60] Liu Y, Li X, Li K, Liu H, Lin C. Multiple bHLH proteins form heterodimers to mediate CRY2-dependent regulation of flowering-time in *Arabidopsis* [J]. PLoS Genet. 2013;9(10). 10.1371/journal.pgen.1003861 PMC3794922 24130508

[61] Cao X, Wen Z, Shen T, Cai X, Hou Q, Shang C et al. Overexpression of *PavbHLH28* from *Prunus avium* enhances tolerance to cold stress in transgenic *Arabidopsis* [J]. BMC Plant Biol. 2023;23(1). 10.1186/s12870-023-04666-1 PMC10726552 38110865

[62] Shen T, Wen X, Wen Z, Qiu Z, Hou Q, Li Z et al. Genome-wide identification and expression analysis of bHLH transcription factor family in response to cold stress in sweet cherry (*Prunus avium* L.) [J]. Sci Hortic. 2021;279.

[63] Sitakanta P, H X C, Ling Y. The interaction domains of the plant myc-like bHLH transcription factors can regulate the transactivation strength [J]. Planta. 2008;227(3). 10.1007/s00425-007-0676-y 18075757

[64] Hou Q, Li S, Shang C, Wen Z, Cai X, Hong Y et al. Genome-wide characterization of chalcone synthase genes in sweet cherry and functional characterization of *CpCHS1* under drought stress [J]. Front Plant Sci. 2022;13. 10.3389/fpls.2022.989959 PMC9437463 36061761

[65] Cheng L, Zhang N, Huang B (2016). Effects of 1-aminocyclopropane-1-carboxylate-deaminase–producing bacteria on perennial ryegrass growth and physiological responses to salinity stress[J]. J Am Soc Hortic Sci.

[66] Liu Y, Liu Q, Li X, Zhang Z, Ai S, Liu C (2023). MdERF114 enhances the resistance of apple roots to Fusarium solani by regulating the transcription of MdPRX63[J]. Plant Physiol.

[67] Livak KJ, Schmittgen TD. Analysis of relative gene expression data using real-time quantitative PCR and the 2(-Delta Delta C(T)) method [J]. Methods (San Diego, Calif). 2001;25(4): 402-8. 10.1006/meth.2001.1262 11846609

