# Supplementary material for: Overexpression of PavHIPP16 from Prunus avium enhances cold stress tolerance in transgenic tobacco
Source: BMC Plant Biol. 2024 Jun 12;24:536. doi: 10.1186/s12870-024-05267-2 (PMC11167810; doi:10.1186/s12870-024-05267-2)
Supplement: Supplementary file 1 — Supplementary Material 1 [file 12870_2024_5267_MOESM1_ESM.docx]

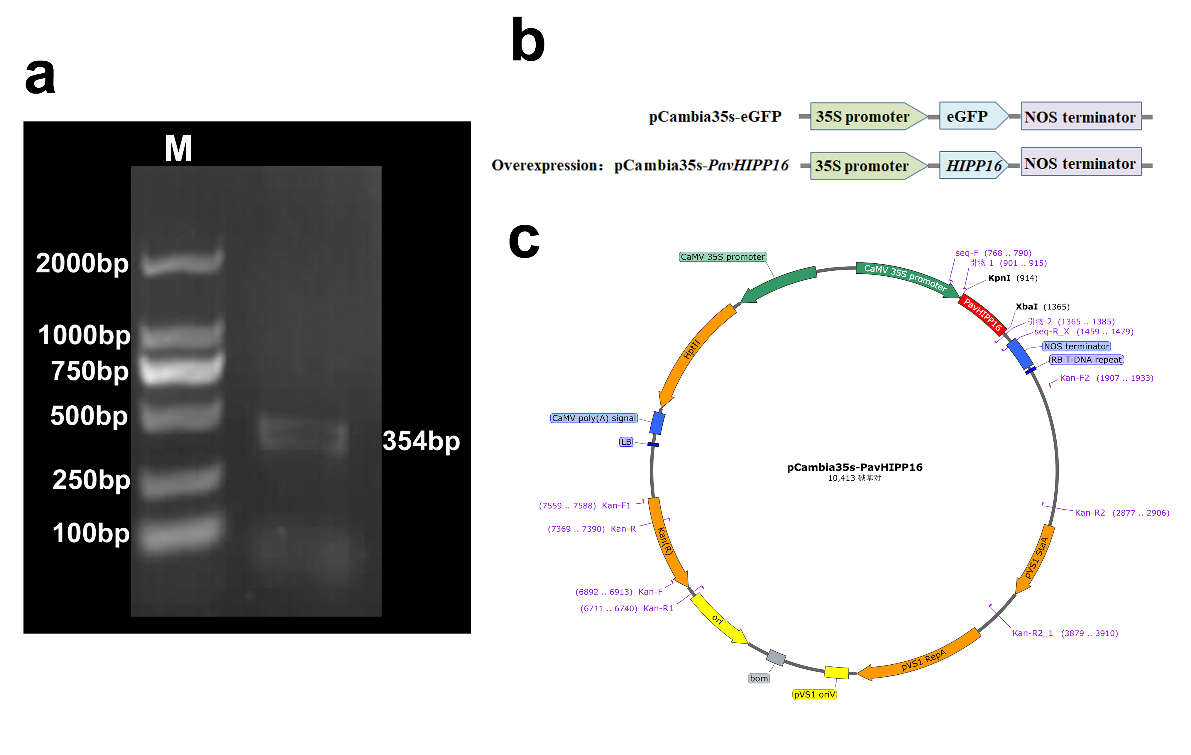
**Figure S1.** **PavHIPP16 gene cloning and overexpression vector construction.**

1. PCR amplification of PavHIPP16 full-length sequence; **(b)** PavHIPP16 overexpression vector construction strategy; **(c)** PavHIPP16 overexpression vector map.


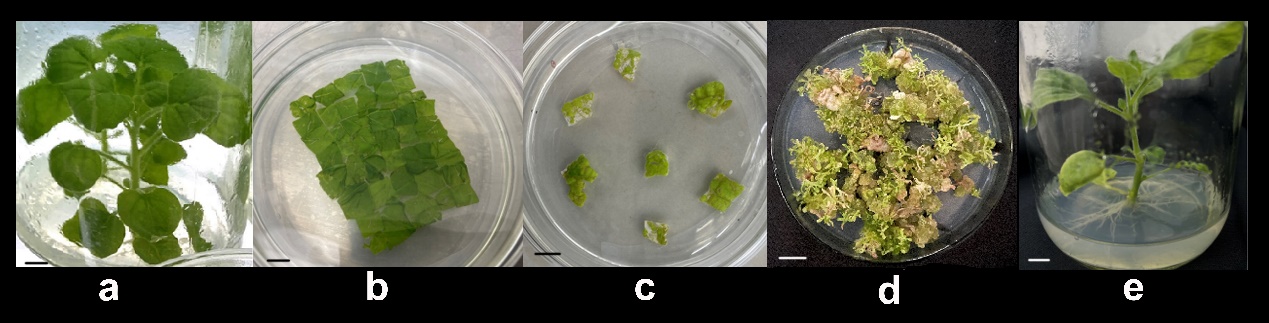


**Figure S2.** ***PavHIPP16* gene genetic transformation of tobacco process.**

**(a)** WT tobacco histocultured seedlings; **(b)** *PavHIPP16*-transformed tobacco co-cultivation stage; **(c)** Screening stage; **(d)** Callus growth of resistant seedlings; **(e)** Transplantation of resistant seedlings to rooting medium
