# Supplementary material for: Overexpression of PavHIPP16 from Prunus avium enhances cold stress tolerance in transgenic tobacco
Source: BMC Plant Biol. 2024 Jun 12;24:536. doi: 10.1186/s12870-024-05267-2 (PMC11167810; doi:10.1186/s12870-024-05267-2)
Supplement: Supplementary file 2 — Supplementary Material 2 [file 12870_2024_5267_MOESM2_ESM.docx]

| **Supplemental Table. The primers used in this study.** | | |
| --- | --- | --- |
| **Annotation** | **Primer name** | **Sequences (5'-3')** |
| Gene cloning | *HIPP16* | F: gatagccatggtacc ATGAAGCAAAAGGTGGTG  R: cagttggaattctagaTTACATAATAGAGCAACCGGG |
| qRT-PCR | *Nt-Actin-* | F: ACTGAAGCGCCTCTTAACCC  R: AGACGGAGAATGGCATGTGG |
|  | *HIPP16-q* | F: GGACAAGGGCCTAATAGAAGTGA  R: TCTTGGTATGGTGAACAGGGAG |
|  | *NtCBF1* | F: CAGGTAAGTGGGTGTGTGAAGT  R: TGCGATCTCGGCTGTTAGG |
|  | *NtCBF2* | F: GTTGAAGTCTTCCTGCCGTC  R: GACTCCACTGGATACCTCGG |
|  | *NtCOR47* | F: TGTCATCGAAAAGCTTCACCGA  R: ACCGGGATGGTAGTGGAAACTG |
|  | *NtCOR78* | F: CGTTTGCTCCAAGTGGTGAT  R: TTCACTTCCTTACCGAGAACA |
|  | *NtFT* | F: AAGCCCAAGCAACCCTAACCTG  R: TGTGCTGAAATTCTGACGCCAACCTG |
|  | *NtCO* | F: GTCACCACCGTGTCCCAATTATGCC  R: ACTTCCCCACCAAACAACATCCCAT |
|  | *NtSOC1* | F: CTCTAAACGCAGAAATGGTTTGCT  R: ATGCTGCAGATTCTGTTCCCC |
|  | *NtFUL* | F: ACTTTCTCAAAACGTCGATCTGGT  R: CTGCAAAACCTCAAGTCTAGCCT |
|  | *NtLFY* | F: AGCATCCTTTTATCGTGACGGAGC  R: CCCTGCCTTCTTCGCGTACCTG |
| Y2H analysis | PavbHLH106-BD | F: catggaggccgaattATGCAGCCAGCTGATC  R: cgctgcaggtagaTTAAAGGACTAATGTGCGG |
|  | PavHIPP16-AD | F: ccgcctcggcctctagaATGAAGCAAAAGGTG  R: cagctcgagctcgatggatccTTACATAATAGAGCAAC |
| Luciferase complementation assay | PavbHLH106-cLUC | F: cccggggcggtaccATGCAGCCAGCTGATC  R: agtagtcgatttgttggatccAAGGACTAATGTGCGG |
|  | PavHIPP16-nLUC | F: cgagaagctcgagtagtcgacATGAAGCAAAAGGTGG  R: cgccccgggacgcgtCATAATAGAGCAAC |

Lowercase letters refer to nucleotide sequences in the vector.
