# Supplementary figures and images for: Overexpression of PavHIPP16 from Prunus avium enhances cold stress tolerance in transgenic tobacco
Source: BMC Plant Biol. 2024 Jun 12;24:536. doi: 10.1186/s12870-024-05267-2 (PMC11167810; doi:10.1186/s12870-024-05267-2)

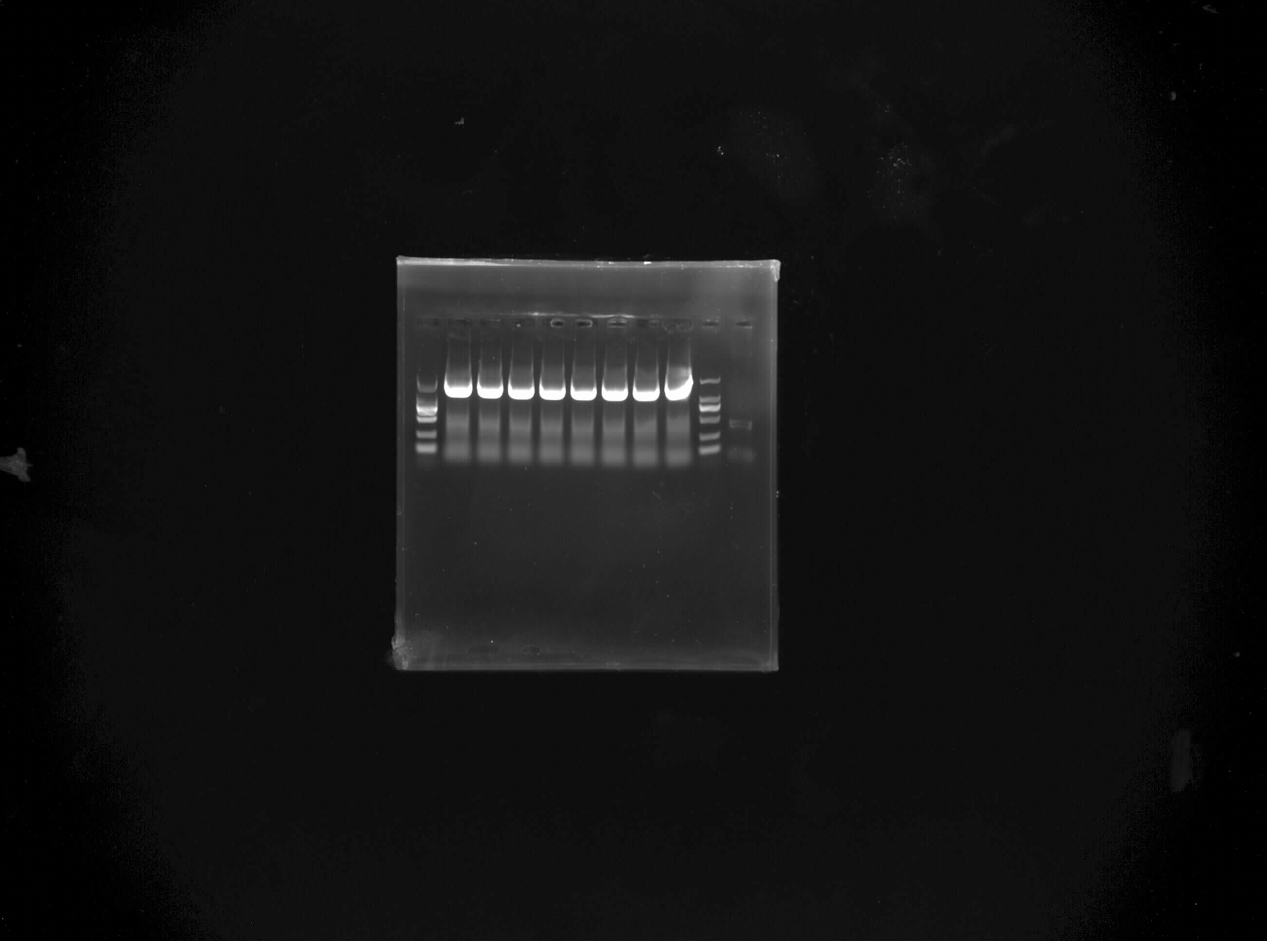


*PavHIPP16* clone gel images


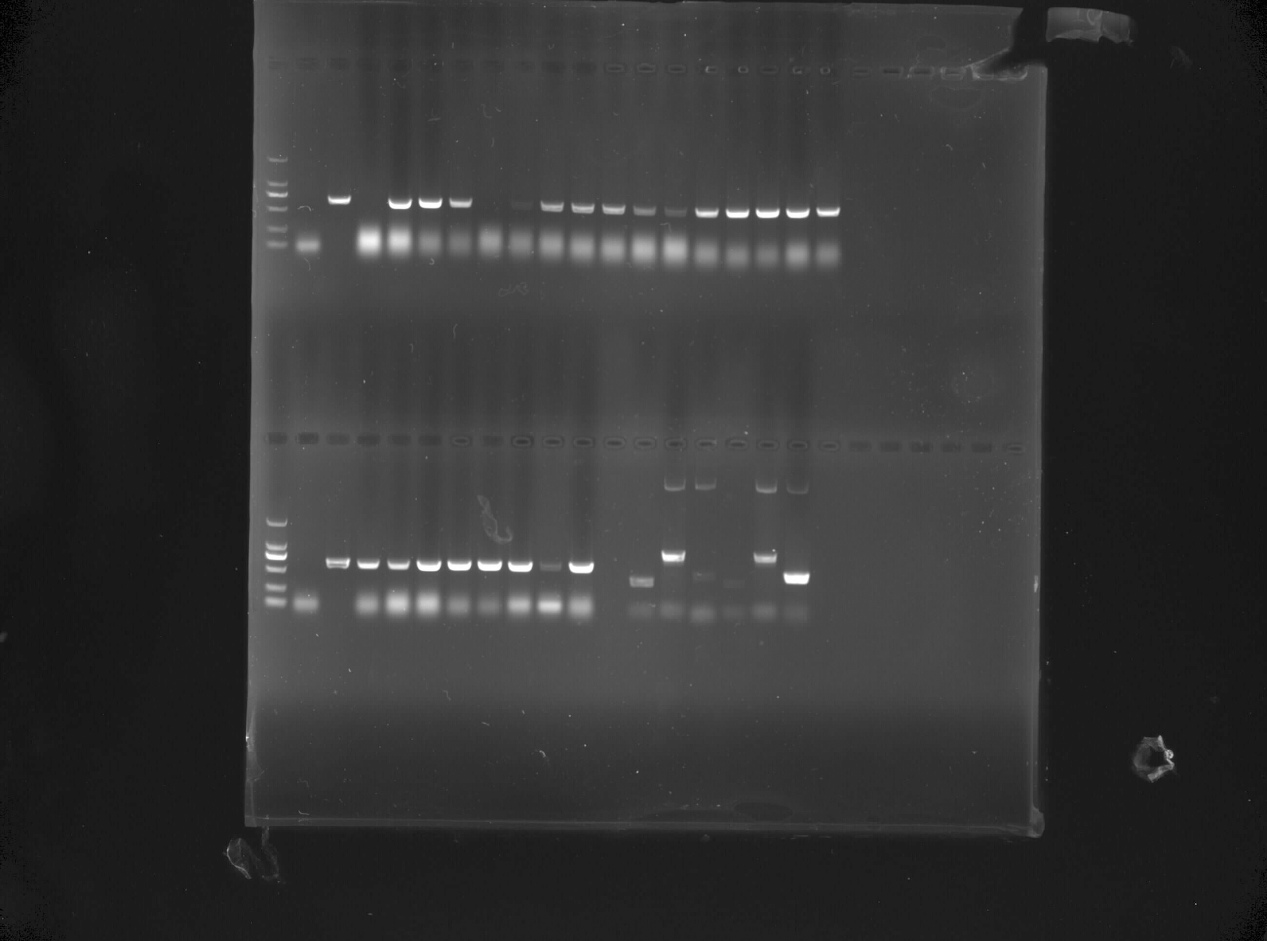


Overexpression of *PavHIPP16* tobacco-positive validation gel image

Supplement: Supplementary file 3 — Supplementary Material 3: An additional movie file shows this in more detail [see Additional file 1: Supplemental Table] [file 12870_2024_5267_MOESM3_ESM.docx]
